# Supplementary material for: Modulation of Pulmonary Fibrosis by Pulmonary Surfactant-Associated Phosphatidylethanolamine In Vitro and In Vivo
Source: Int J Mol Sci. 2025 Jul 24;26(15):7132. doi: 10.3390/ijms26157132 (PMC12345749; doi:10.3390/ijms26157132)
Supplement: Supplementary file 1 [file ijms-26-07132-s001.zip › ijms-3696525-supplementary.pdf]

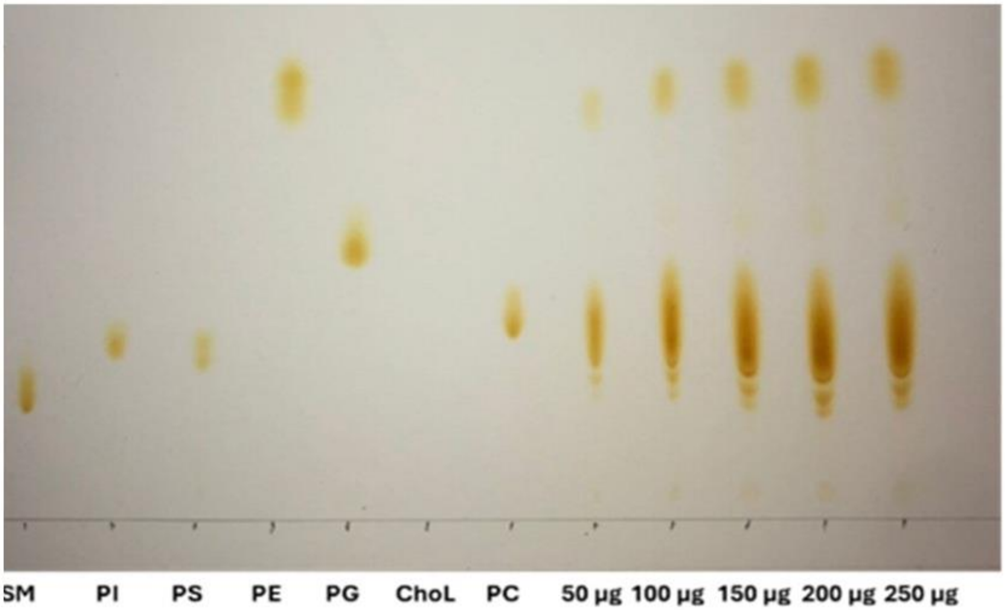

Figure S1. Determination of SPNPC lipids by thin layer chromatography (TLC). The plate reveals the presence of sphingomyelin (SM), phosphatidylinositol (PI), phosphatidylserine (PS), phosphatidylethanolamine (PE), phosphatidylglycerol (PG), cholesterol (CHOL), phosphatidylcholine (PC) and gradual concentrations of SPNPC
